# Meet the Section Editor

**DOI:** 10.2174/1570159X2209240229142958

**Published:** 2024-02-29

**Authors:** Gelsomina Mansueto

**Affiliations:** 1 Department of Advanced Clinical and Surgical Sciences, University of Campania “Luigi Vanvitelli”, Naples, Italy

   **Prof. Gelsomina Mansueto** graduated in medicine and surgery from the University of Naples “Federico II” in 1997. She specialized in pathological anatomy and cytopathology in 2003, and obtained a doctorate of research in clinical and pathological morphology from the same University of Naples “Federico II” in 2006. In 2020, she became the associate professor in general and clinical pathology in the Department of Advanced Medical and Surgical Sciences at the University of Campania “Luigi Vanvitelli”, Italy. She has experience in tumor and inflammatory histopathology, especially cardiopathology, neuropathology, and sudden death. She taught and did research activity in pathological anatomy, forensic medicine and clinical pathology at the University of Naples Federico II and University of Campania L. Vanvitelli, Italy. She is the author and co-author of over 100 scientific works. Her major fields of interest are stroke histology, neurodegenerative diseases, cardiovascular and pulmonary pathology, molecular and immunohistochemical markers of vitality, acute and chronic asphyxia, cardiac malformations and infectious diseases.
